# Draft Genome Sequence of MCPA-Degrading Sphingomonas sp. Strain ERG5, Isolated from a Groundwater Aquifer in Denmark

**DOI:** 10.1128/genomeA.01529-14

**Published:** 2015-02-12

**Authors:** Tue Kjærgaard Nielsen, Witold Kot, Sebastian R. Sørensen, Lars Hestbjerg Hansen

**Affiliations:** a Department of Environmental Science, Aarhus University, Roskilde, Denmark; b Department of Biology, Faculty of Science, University of Copenhagen, Copenhagen, Denmark; c Department of Geochemistry, Geological Survey of Denmark and Greenland (GEUS), Copenhagen, Denmark

## Abstract

Sphingomonas sp. strain ERG5 was isolated from a bacterial community, originating from a groundwater aquifer polluted with low pesticide concentrations. This bacterium degrades 2-methyl-4-chlorophenoxyacetic acid (MCPA) in a wide spectrum of concentrations and has been shown to function in bioaugmented sand filters. Genes associated with MCPA degradation are situated on a putative conjugative plasmid.

## GENOME ANNOUNCEMENT

Herbicides such as 2-methyl-4-chlorophenoxyacetic acid (MCPA) are widely used for crop protection across the world, with the implication that they frequently occur in groundwater reservoirs, for example, in the European Union, where they are threatening an important and sensitive source of freshwater (1).

The MCPA-degrading bacterium Sphingomonas sp. strain ERG5 was previously isolated from an enriched bacterial community that originated from an aquifer located below a conventionally treated agricultural field at Fladerne Creek in Denmark (2). This strain can readily mineralize MCPA at both low and high concentrations (10 µg·L^−1^ to 10 mg·L^−1^), making it a strong candidate for bioremediation purposes (3). Sphingomonas sp. ERG5 was previously sequenced, in order to investigate the genes encoding the degradation pathway. It was discovered that the entire putative pathway is encoded within an approximately 33-kbp transposon, which in turn is placed on a 138-kbp putative plasmid that also harbors the genes associated with conjugative transfer via the type 4 IV secretion system, as well as multiple plasmid stability genes (4). The Sphingomonadaceae family is often associated with degradation of xenobiotics, and several strains have been isolated from polluted environments and have been shown to have their degradative genes situated on large plasmids (5). Members of this family appear to often harbor conjugative plasmids that are rarely transferred to other bacterial families (6).

Sphingomonas sp. ERG5 was streaked on an R2A plate (Sigma-Aldrich, St. Louis, MO, USA) and incubated at 20°C for 4 days. A single colony from this plate was picked for DNA extraction using an Ultra-Clean Microbial DNA isolation kit (MoBio Laboratories, Inc., Carlsbad, CA, USA). Genomic DNA was prepared for paired-end sequencing on the Illumina MiSeq platform using the Nextera XT DNA sample preparation kit (Illumina, San Diego, CA, USA). Libraries were sequenced using the MiSeq version 2 reagent kit (Illumina), yielding 503,739 read pairs for an estimated 44-fold coverage of the genome. Sequencing adapters were removed with Cutadapt (7), and assembly was performed with the SPAdes version 3.1 genome assembler (8) with the “careful” option enabled and all other options set to default. Assembly was evaluated using QUAST (9), and contigs smaller than 200 bp were removed. The final assembly has 86 contigs, of which 80 are larger than 1,000 bp, totaling 5,739,859 bp with a G+C content of 63.73% and an *N*_50_ of 124,832. The draft genome was automatically annotated using RAST (10), which predicted 5,561 coding sequences and 51 RNA-encoding genes. The 16S rRNA gene of Sphingomonas sp. ERG5 is 99% similar to Sphingomonas alpina strain S8-3 (accession no. GQ161989.1). The previously described plasmid pCADAB1 that harbors genes associated with the pathway for MCPA degradation (4) is represented by three contigs that are 104,803 bp, 31,433 bp, and 1,266 bp long and are 100% similar to the 138,306 bp pCADAB1.

### Nucleotide sequence accession numbers. 

This whole-genome shotgun project has been deposited at DDBJ/EMBL/GenBank under the accession number JSXI00000000. The version described in this paper is the first version, JSXI01000000.
